# P-335. Environmental Impact of Contact Precaution Use for Methicillin-resistant *Staphylococcus aureus* (MRSA) and Vancomycin-resistant *Enterococcus* (VRE) in Los Angeles (LA) County: Results of a Countywide Survey

**DOI:** 10.1093/ofid/ofae631.538

**Published:** 2025-01-29

**Authors:** Pam Lee, Kelsey OYong, Michelle LeBrun, Zachary A Rubin, Loren G Miller

**Affiliations:** Harbor UCLA, Long Beach, California; Los Angeles County Department of Public Health, Los Angeles, California; Harbor UCLA, Long Beach, California; Los Angeles County Department of Public Health, Los Angeles, California; Lundquist Institute at Harbor-UCLA Medical Center, Los Angeles, CA

## Abstract

**Background:**

United States hospitals produce over 5.9 million tons of waste yearly. Disposable personal protective equipment (PPE) causes up to 70% of hospital municipal plastic waste. Contact precautions, often requiring disposable gowns and gloves, are frequently used to prevent MRSA/VRE transmission. We determined the scope and environmental impact of contact precaution use for MRSA and VRE in LA County.

**Methods:**

We observed patient care at LA acute care hospitals to quantify PPE used for patients in MRSA/VRE contact precautions, as well as for patients with methicillin-susceptible *Staphylococcus aureus* (MSSA) and vancomycin-susceptible *Enterococcus* (VSE). We matched observations by hospital type (community, academic safety-net) and care location (intensive care, telemetry, ward). We conducted 24 observations for each organism.

To determine the extent of PPE used for MRSA/VRE, the LA County Department of Public Health surveyed acute care hospitals for the average daily number of hospitalized patients in 2023 in MRSA/VRE contact precautions. We modeled the county-wide solid waste and carbon dioxide (CO_2_) emissions from MRSA/VRE contact precautions.

**Results:**

Mean gown use was higher for MRSA vs. MSSA (2.33 vs. 0.17 gowns/hour, p< 0.01) and VRE vs. VSE (2.50 vs. 0.59 gowns/hour, p< 0.01). Glove use did not vary between MRSA vs. MSSA (5.08 vs. 4.33 gloves/hour, p = 0.45) or VRE vs. VSE (5.88 vs. 5.46 gloves/hour, p = 0.50).

In total, ∼3% of LA County hospitals’ licensed beds are occupied by patients in contact precautions for MRSA and VRE. In our model this level of occupancy equaled 7.3 million gowns per year for MRSA and VRE contact precautions, which generate 461,400 kilograms (kg) of plastic waste and 2.27 million kg of CO_2_ emissions annually.

**Conclusion:**

We found that care for patients in MRSA/VRE contact precautions requires significantly more gown use than for patients with MSSA/VSE. Glove use did not differ between patients with antibiotic-resistant infections vs. their antibiotic-sensitive counterparts. LA County’s MRSA/VRE PPE causes abundant plastic waste and greenhouse gas emissions equal to 5.8 million miles driven in an average car. Routine MRSA/VRE contact precautions need to be reconsidered given the paucity of supporting evidence as well as their potential harm to the environment.

**Disclosures:**

**Loren G. Miller, MD MPH**, Armata: Grant/Research Support|Contrafect: Grant/Research Support|GSK: Grant/Research Support|Merck: Grant/Research Support|Paratek: Grant/Research Support

